# Supplementary material for: An rRNA hybridization-based approach for rapid and accurate identification of diverse fungal pathogens
Source: Microbiol Spectr. 2026 Sep 25;14(11):e02696-26. doi: 10.1128/spectrum.02696-26 (PMC13729197; doi:10.1128/spectrum.02696-26)
Supplement: Supplementary file 1 [file spectrum.02696-26-s0001.pdf]

Supplementary Information

A rRNA hybridization-based approach for rapid and accurate identification of diverse fungal pathogens

Elizabeth A. Yee[1*], Brooks J. Burt[2*], Poppy C. S. Sephton-Clark[2,3], M. Lauren Donnelly-Morell[1,4], Aidan Z. Wang[1], Isaac H. Solomon[5], Evan Mojica[7], Christina A. Cuomo[1, 6#], Roby P. Bhattacharyya[1, 8#]

[1]Infectious Disease and Microbiome Program, Broad Institute of MIT and Harvard, Cambridge MA 02142 USA
[2]Genomic Center for Infectious Disease, Broad Institute of MIT and Harvard, Cambridge MA 02142 USA
[3]Present address: Valo Health, 75 Hayden Avenue Suite 200, Lexington, MA 02421 USA
[4]Department of Pathology, Massachusetts General Brigham, Harvard Medical School, Boston, MA USA
[5]Department of Pathology, Brigham and Women's Hospital, Harvard Medical School, Boston, MA 02115 USA
[6]Department of Molecular Microbiology and Immunology, Brown University, Providence RI 02912 USA
[7]Microbiology Laboratory, Department of Pathology, Massachusetts General Hospital, Boston, MA 02114, USA
[8]Infectious Diseases Division, Department of Medicine, Massachusetts General Hospital, Boston MA 02114 USA
*Denotes Co-First authorship: EAY and BJB contributed equally.
[#]To whom correspondence should be addressed

Supplementary Information: Contents

p. 1 Index
p. 2-3 Supplementary Methods
p. 4-14 Supplementary Figures S1-S10
p. 15-16 Supplementary References
(note: Supplementary Tables available as a separate file)

Supplementary Methods

Sample Acquisition

Isolates only able to be identified to genus level in the clinical microbiology laboratory included all *Curvularia*, *Coccidioides*, *Fusarium*, *Cunninghamella*, *Mucor*, *Rhizopus* and *Talaromyces* isolates. Outside of samples acquired from our own laboratory and the ARBank, additional samples were generously shared with us from the following sources: *Histoplasma capsulatum* (n = 3) cell pellets suspended in Trizol from Dr. Sinem Beyhan (JCVI) (1); *Pneumocystis jirovecii* gDNA from human autopsy samples from Dr. Joseph Kovacs (NIH) (n = 3); and *Blastomyces dermatitidis* total RNA, as well as gDNA from *Cryptococcus gattii, Paracoccidioides brasiliensis, Sporothrix schenckii* and *Talaromyces marneffei* from the Fungal Genomics Group at the Broad Institute (n = 19) (1–5).

Coded/de-identified FFPE tissue samples (n = 27) were obtained from the Brigham & Women's Hospital (BWH) Pathology Department, after visual screening to identify archived clinical samples from 2019-2025 with varying levels of visible fungal forms (ranging from rare to confluent). Fungal identification of these autopsy and surgical pathology cases were based on integration of all available laboratory results from anatomic pathology histochemical stains, culture isolation from concurrently collected tissue samples, and molecular testing of FFPE tissue, frozen tissue, or culture isolates (6). Supplementary Table S3d contains additional information on each FFPE sample.

Probe Design

Reference Sequences: 18S and partial 28S sequences for the 86 ingroup species were primarily obtained from the Silva database (7). Where Silva sequences for target species were too short, or unavailable, regions were retrieved from NCBI assemblies for the following

species: *Mucor circinelloides*, *Emergomyces orientalis*, *Epidermophyton floccosum*, *Coccidioides posadasii*, *Coccidioides immitis*, *Histoplasma capsulatum*, *Emergomyces pasteurianus*, *Exophiala dermatitidis*, *Trichophyton indotiniae*, *Kodamaea ohmeri*, *Wickerhamomyces anomalus*, *Kluyveromyces marxianus*, *Malassezia dermatis*, *Enterocytozoon bieneusi*. All retrieved sequences were validated using an alignment of each gene and manual inspection. While 18S sequences appeared largely complete, due to inconsistent annotation of 28S sequences, full-length 28S sequences could not be obtained for all species.

Design rationale for species and taxon-level probes: For species-specific probe design, an outgroup of all other pathogenic and environmental fungi was provided to optimize specificity (8). This approach aimed to design probes predicted to bind uniquely to the targeted species in a given rRNA region (Table S2). Probes were also designed to uniquely target higher-level taxa (genus, family, order, or class), including all members of that taxon but no non-members, across both pathogens and environmental fungi. The inclusion of non-pathogens provided necessary sequence diversity to identify taxonomic probes.

Control probes: The 6 positive control probes target External RNA Controls Consortium (ERCC) spike-ins included in every hybridization reaction at pre-specified concentrations; the 6 negative control probes target bioorthogonal sequences not present in the hybridization. All control probes are provided as part of standard NanoString hybridizations (9).

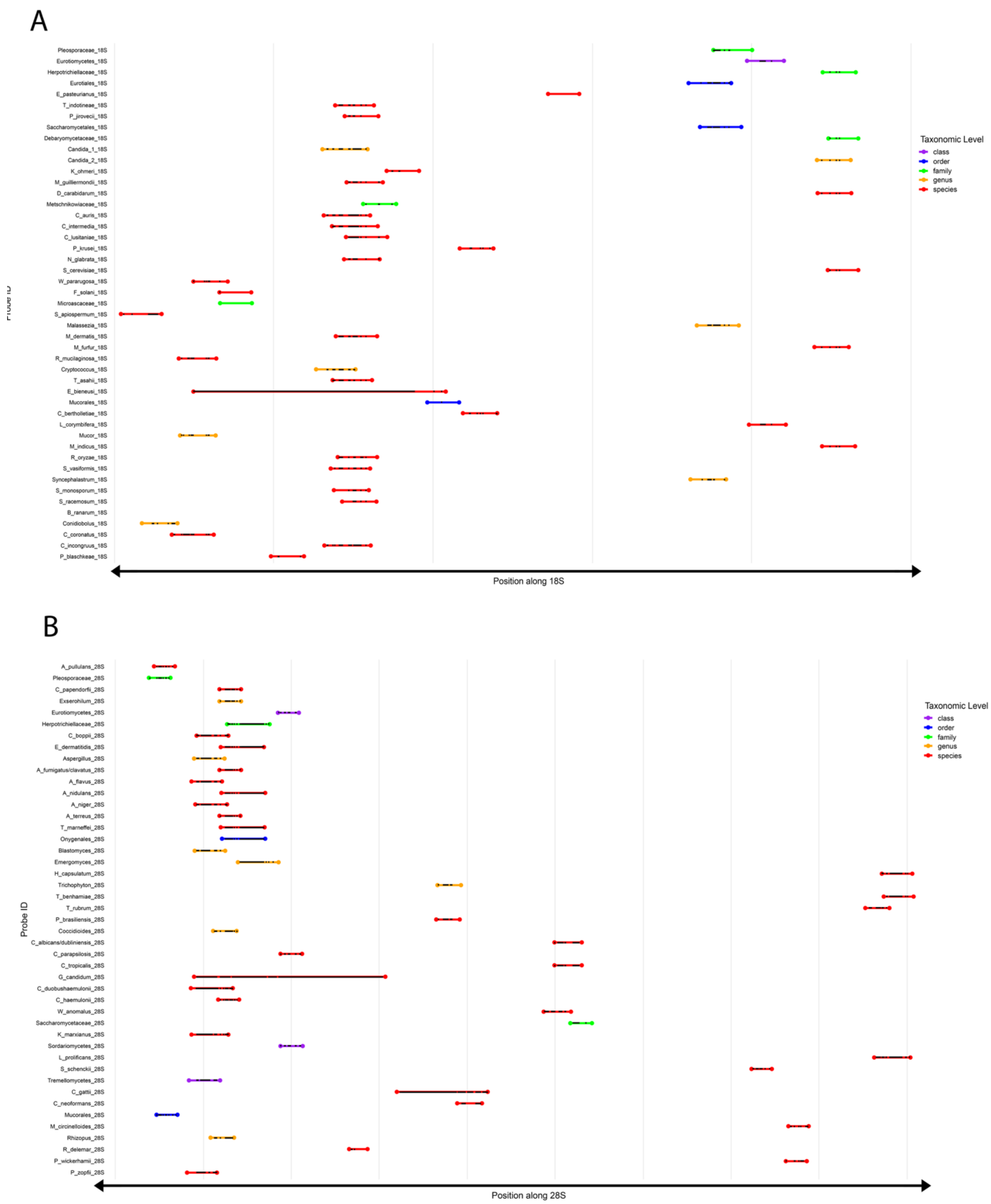

Figure S1. Coverage maps of Pan-Fungal Phirst-ID probe sequences along 18S and 28S rRNA subunits. Probe sequences mapped along target rRNA sequence of (A) 18S and (B) 28S subunits. Color of line indicates taxonomic level specificity of probe. Longer mappings indicate gaps in alignment due to genetic variation in the consensus sequence. Light gray vertical lines indicate 500 bp distance along the consensus 18/28S axes (including gaps). Probe sequences are available in Supplementary Table S2.

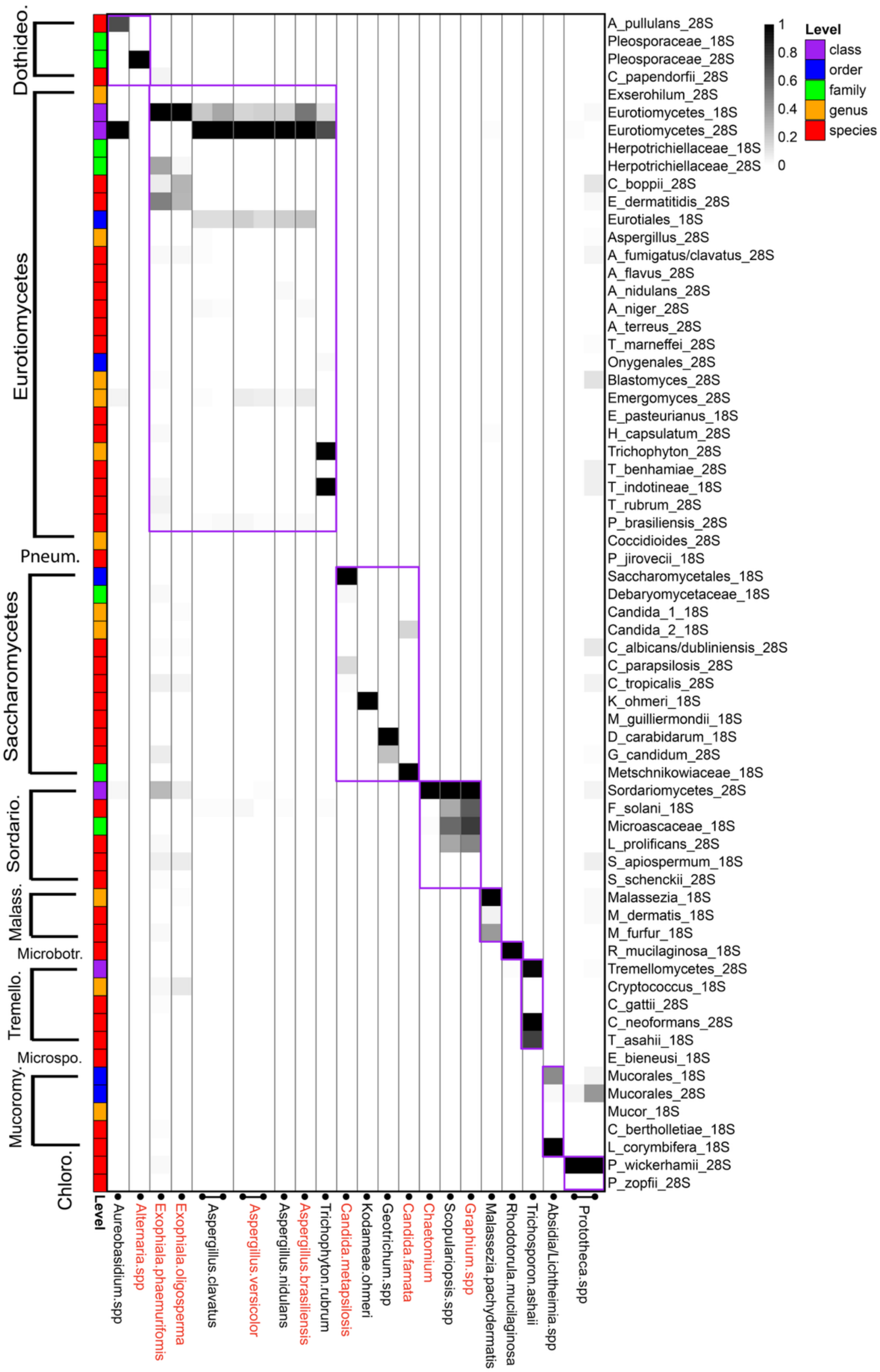

Figure S2 Pan-Fungal Phirst-ID PSRPs from the Underrepresented Set. Heatmap displaying normalized probe binding intensities of 24 additional samples, for which we were only able to obtain 1 or 2 examples and thus did not meet criteria for inclusion in the Training or Validation Sets (because we could not rigorously apply our sample identification algorithms). Purple boxes indicate regions where the class of samples and intended probe targets match. Red text indicates species not included in probeset design but obtained for testing.

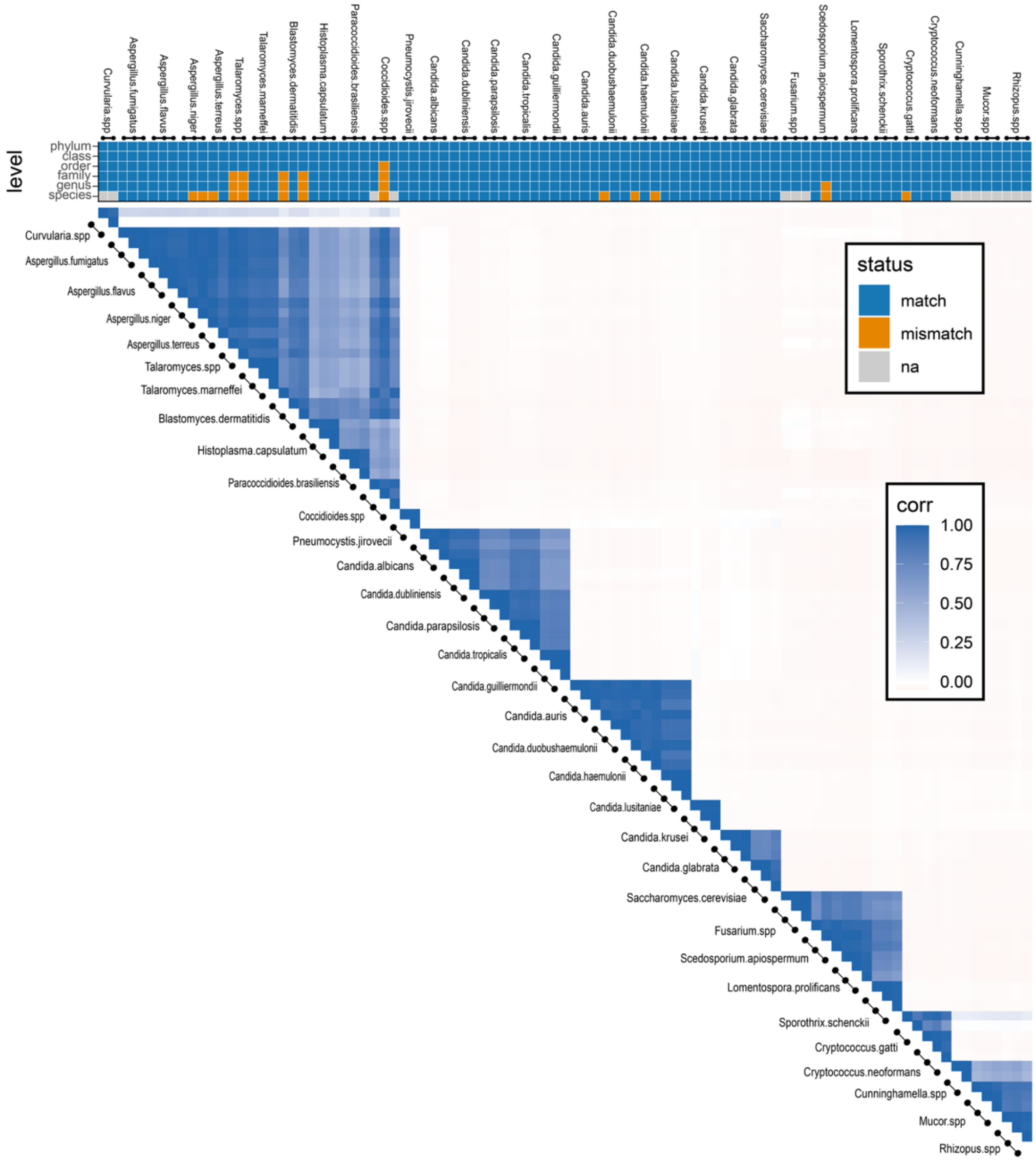

Figure S3. Pearson correlations of Training Set PSRPs are highest between closely related species. Samples with similar taxonomic classification had higher pairwise Pearson correlations than those that were more dissimilar. **(A)** Tile plot indicates prediction accuracy for the single best-matched (highest) non-self-Pearson correlation at each taxonomic level, as in Fig 3a. **(B)** Heatmap of pairwise Pearson correlations, showing that the highest correlations map to members of closely related species. Training Set samples are ordered by taxonomic classification as in Fig 2.

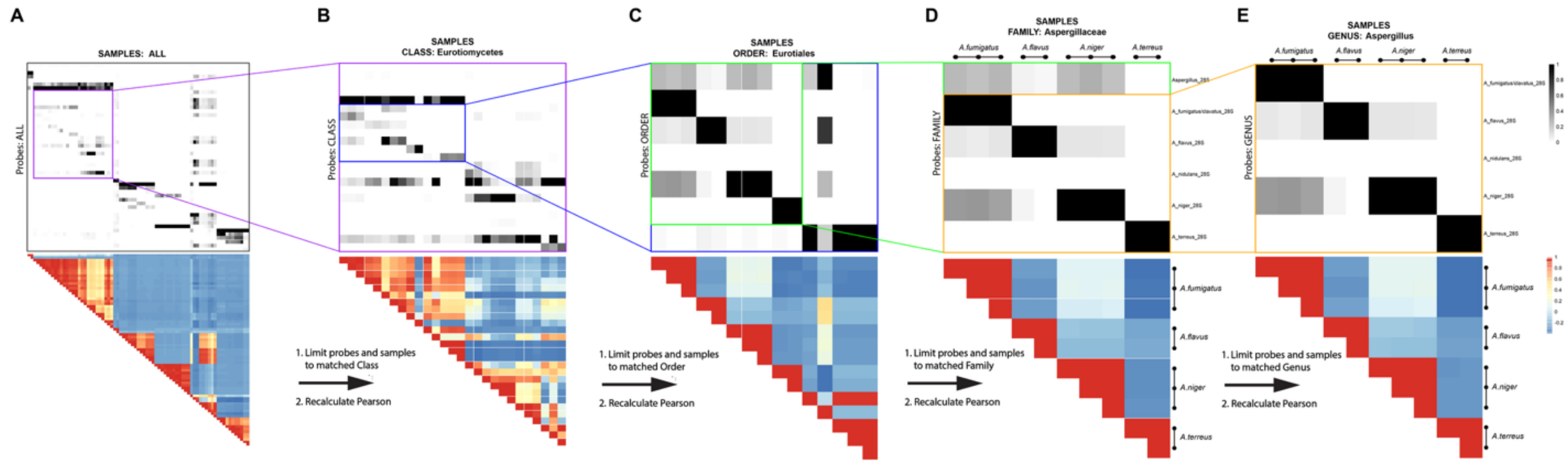

Figure S4. Schematic of our hierarchical Pearson correlation classifier Co-PILOT. Visual representation of how Co-PILOT moves through taxonomic levels, demonstrated on an *Aspergillus fumigatus* sample. Co-PILOT begins by considering all probes and samples **(A)** and chooses the best-matched class based on the class of the single (non-self) sample with the highest Pearson correlation to the query sample. It then restricts further analysis to only samples from that class, and only probes nested within that class (i.e., those designed to target regions matching an order, family, genus, or species within the selected class), indicated by the box (colored by taxonomic level, as in other figures; here, purple box = class); data from all other samples and probes are removed at this stage **(B)**, including probe(s) matching selected class. In subsequent steps, Co-PILOT selects only the subset of samples & probe(s) containing the best-matched order (**B → C**, blue box), family (**C → D**, green box), and genus (**D → E**, orange box). Top panels = read count heatmaps, rescaled by sample at each step to maximize remaining probe; bottom panels = Pearson correlation heatmaps only using selected data at each step.

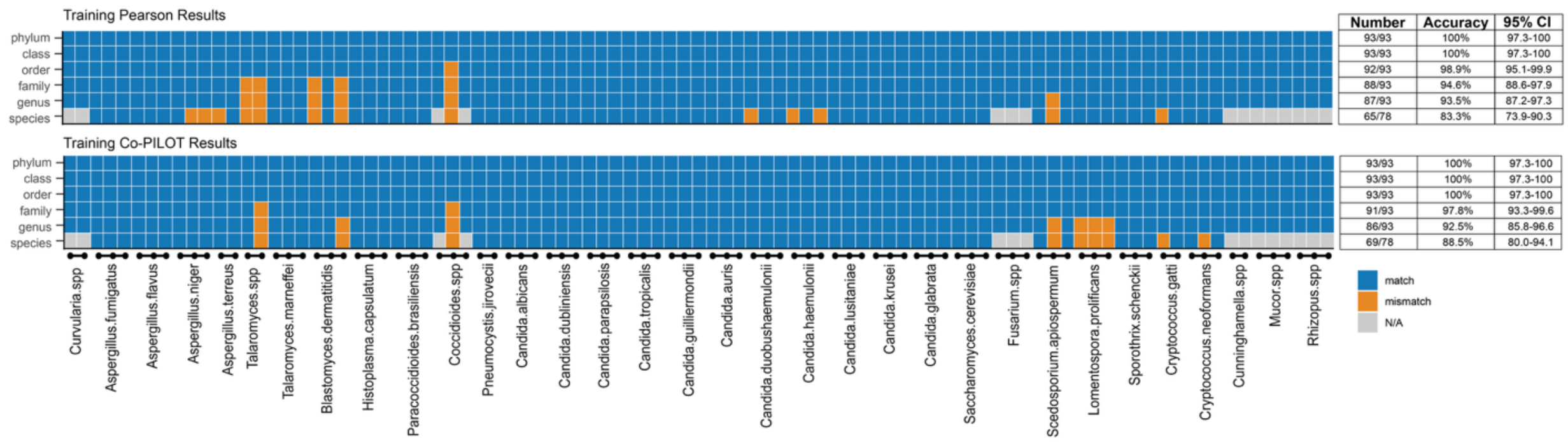

Figure S5. The Co-PILOT classifier improved identification accuracy of the Pan-Fungal Phirst-ID probeset on the Training Set. Tile plot indicating prediction accuracy at each taxonomic level, as in Fig 3a. Co-PILOT (bottom panel) improved accuracy relative to simple Pearson correlations (top panel), though it was iteratively designed based on the Training Set, so these results are best viewed as “overtrained” - see main text and Methods for details. By contrast, its performance on the Validation Set (Fig 6) is an independent assessment of Co-PILOT’s accuracy on data it had not seen until its algorithm (Supp Fig S4) was fully designed.

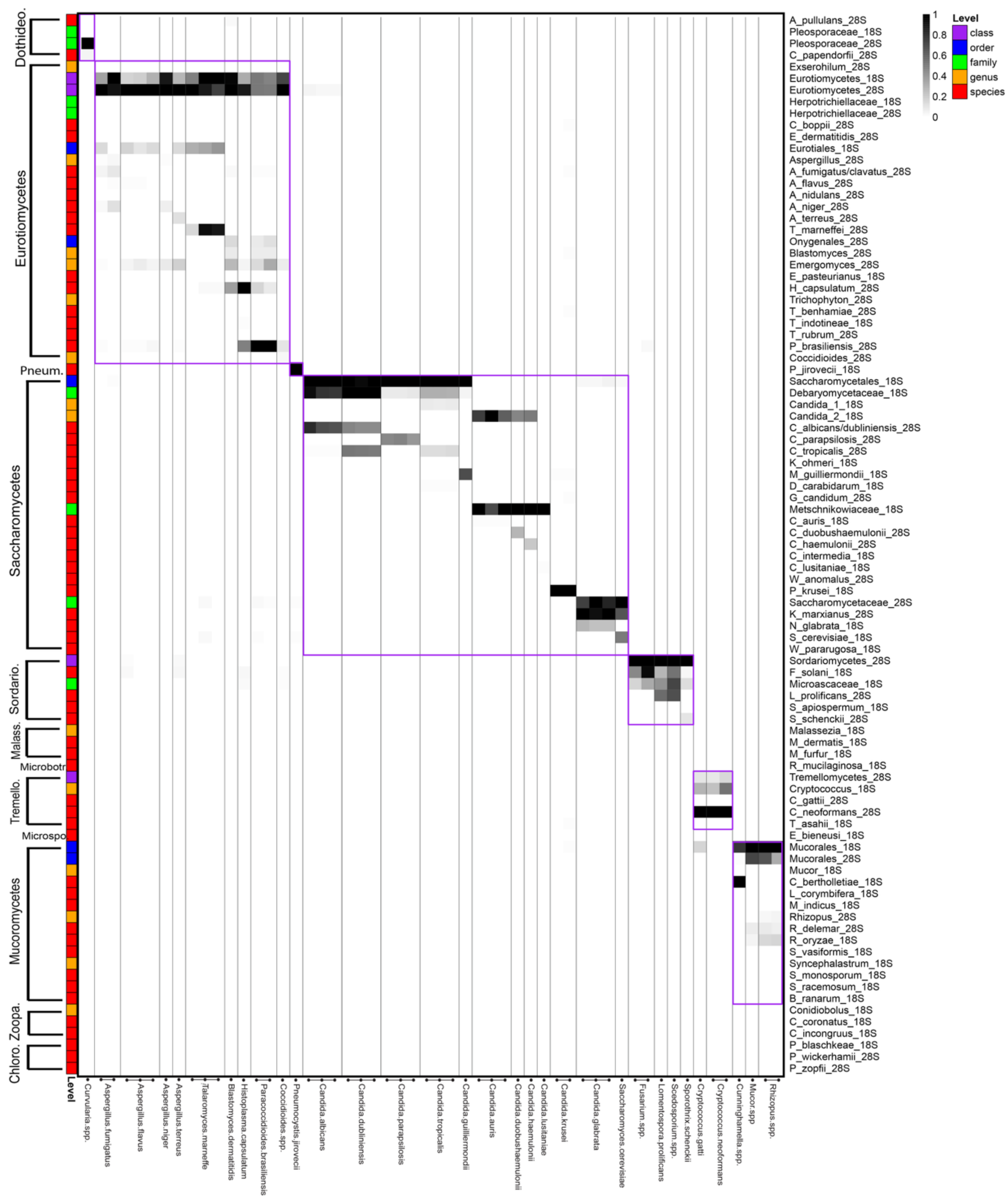

Figure S6. Pan-Fungal Phirst-ID panel generates unique PSRPs for 32 clinically relevant species in our Validation Set. Heatmap of scaled binding intensities of the 54 samples comprising our Validation Set. As in Figure 2, samples and probes are ordered taxonomically, though the cladogram is omitted here. Taxonomic level of each probe is indicated by colored box at left; probe name is listed at right. Purple boxes indicate regions where the class of samples and intended probe targets match.

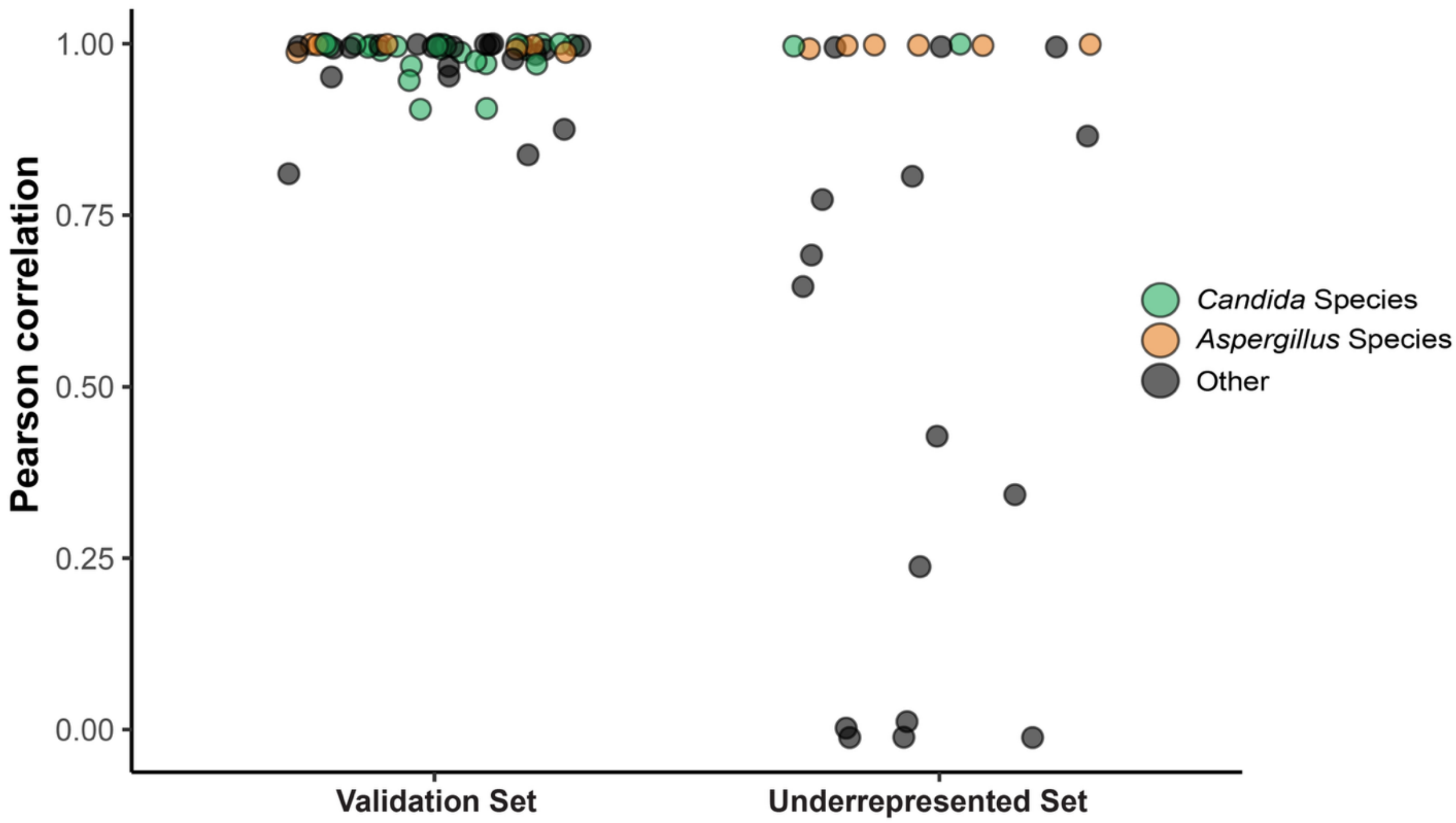

Figure S7. Pearson correlations of best match to Training Set for previously seen and unseen species. Dotplot showing Pearson correlations of test samples against the Training Set reference profile. Samples are grouped by whether their species is represented in the Training Set. The Validation Set (left) contains samples present in the Training Set, whereas the Underrepresented Set (right) contains samples with no matching species. Point color denotes taxonomic groups highlighting *Candida* and *Aspergillus* species in each Set.

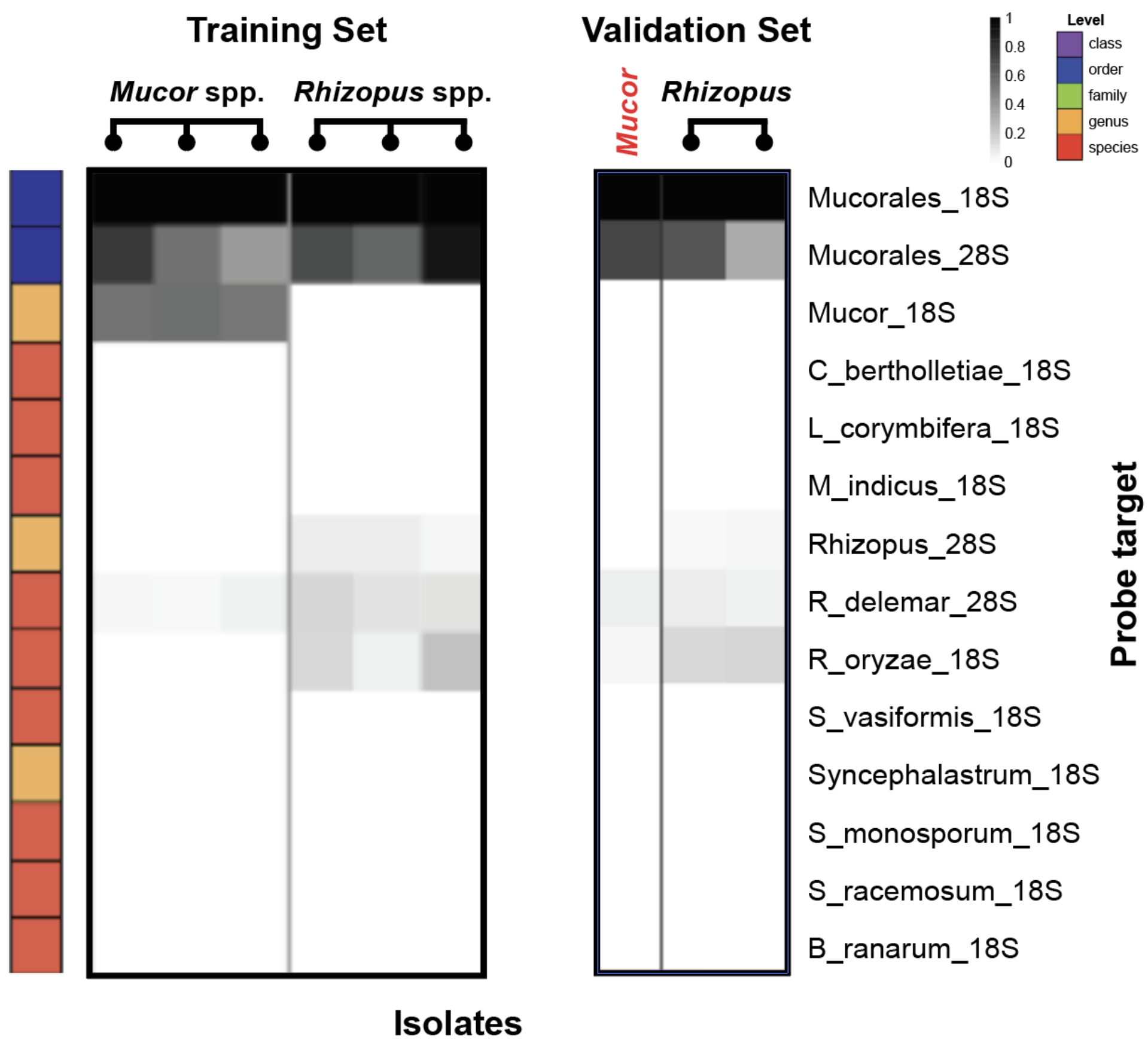

Figure S8. Errant clinical identification of *Mucor* from Validation Set. Pan-Fungal Phirst-ID data from all probes targeting subsets of the *Mucoromycetes* class, shown for all samples from the Training and Validation Sets identified by the clinical microbiology laboratory as either *Mucor* or *Rhizopus*. The red label indicates a Validation sample identified by the clinical laboratory as a *Mucor*, but with a PSRP better resembling a *Rhizopus* (specifically in the lack of Mucor_18S probe reactivity, and perhaps greater R_oryzae_18S probe reactivity). ITS sequencing identified this sample as *Rhizopus microsporus*, more consistent with the Pan-Fungal Phirst-ID prediction than the clinical classification.

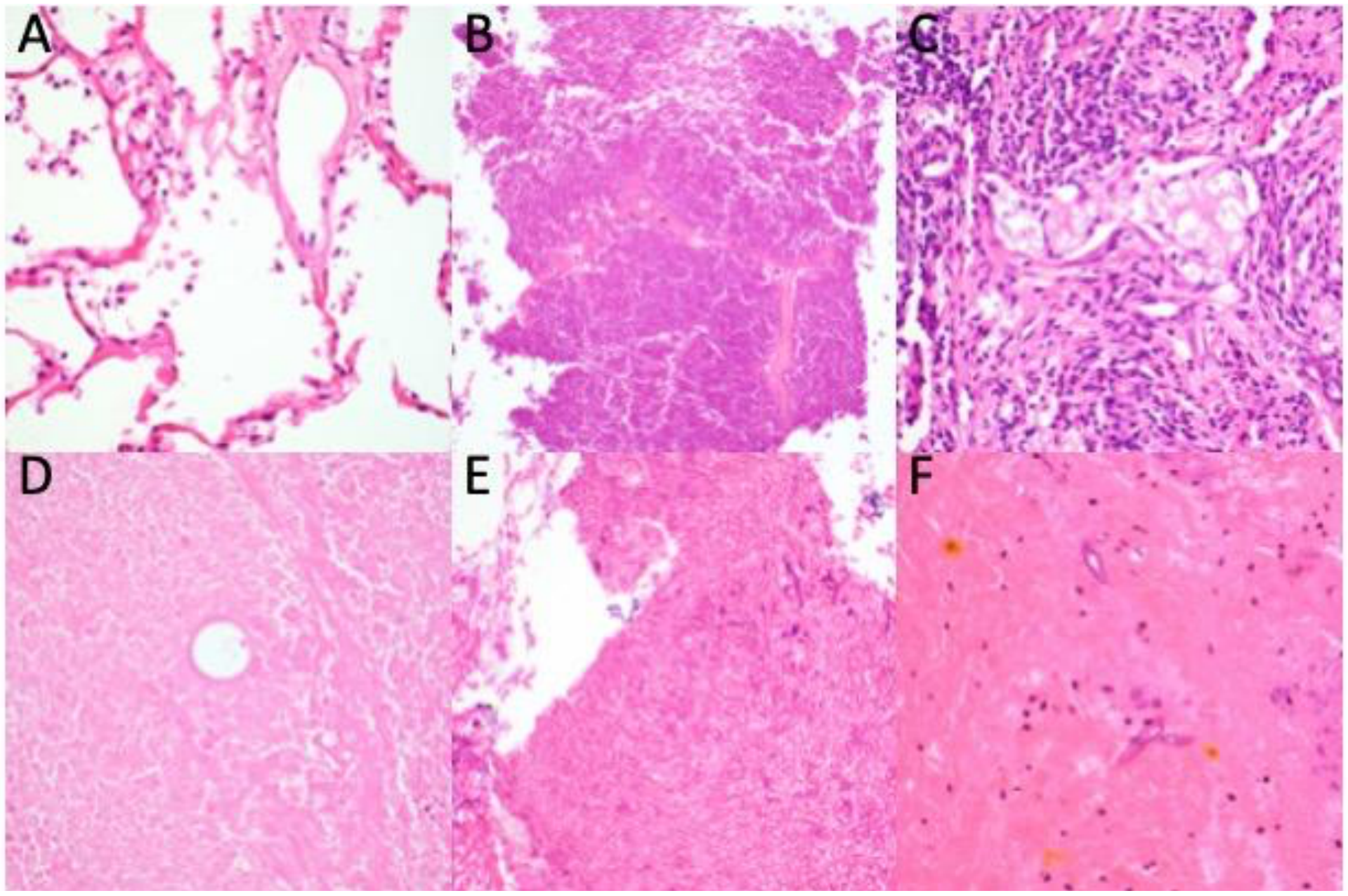

Figure S9. Representative hematoxylin and eosin (H&E)-stained sections from formalin-fixed, paraffin-embedded tissues included in the study. (A) Lung tissue, negative control (Fig S9, right panel, column 11; Supp Table S3d, sample FP002). **(B)** Aortic valve tissue with *Candida parapsilosis* (Fig S9, left panel, column 7; Supp Table S3d, sample FP024. **(C)** Lung tissue with *Cryptococcus neoformans* (Fig S9, right panel, column 8; Supp Table S3d, sample FP011). **(D)** Lung tissue with *Coccidioides* sp (Fig S9, right panel, column 4; Supp Table S3d, sample FP019. **(E)** Sinus tissue with *Aspergillus fumigatus* (Fig S9, left panel, column 5; Supp Table S3d, sample FP028). **(F)** Sinus tissue with *Rhizopus* sp (Fig S9, right panel, column 15; Supp Table S3d, sample FP023). All images were acquired using a 40× objective.

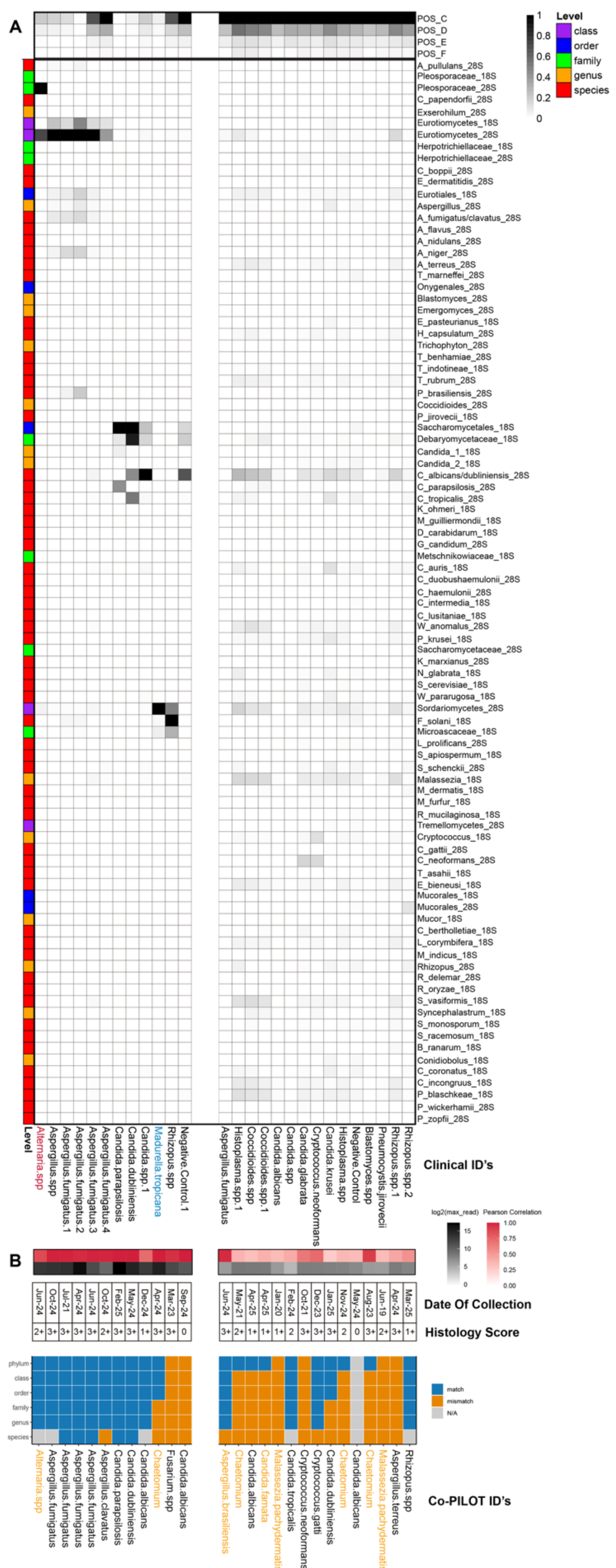

184

Figure S10: Pilot use of Pan-Fungal Phirst-ID for FFPE samples. (A) Heatmap of scaled probe binding intensities of 27 FFPE samples. For column-scaling to better visually represent total read counts, the four lowest-abundance positive control spike-ins are included for each sample (top 4 rows, above horizontal line). Bottom axis indicates clinical identification provided by the Pathology department (see Supplementary Table S3d). Red text indicates an organism not included in probeset design, blue text indicates an organism neither included in probeset design for nor previously tested by Pan-Fungal Phirst-ID (and thus not available to the Co-PILOT algorithm for comparison). **(B)** Tile plot indicating prediction accuracy of Pan-Fungal Phirst-ID with Co-PILOT at each taxonomic level, as in Fig 3a. Date of sample collected is indicated above and final Co-PILOT identification (best match from Training Set) is listed below the tile plot. 12 samples at left, which exceeded our probe detection threshold, are separated from 17 samples at right, which did not. Samples are sorted by taxonomic order of the identification from the clinical microbiology laboratory. Orange text indicates a Co-PILOT prediction of a species not included in probeset design.

Supplementary References

1. Voorhies M, Cohen S, Shea TP, Petrus S, Muñoz JF, Poplawski S, Goldman WE, Michael TP, Cuomo CA, Sil A, Beyhan S. 2022. Chromosome-Level Genome Assembly of a Human Fungal Pathogen Reveals Synteny among Geographically Distinct Species. *mBio* 13:e02574-21.
2. Cuomo CA, Rodriguez-Del Valle N, Perez-Sanchez L, Abouelleil A, Goldberg J, Young S, Zeng Q, Birren BW. 2014. Genome Sequence of the Pathogenic Fungus *Sporothrix schenckii* (ATCC 58251). *Genome Announc* 2:e00446-14.
3. Sephton-Clark P, Nguyen T, Hoa NT, Ashton P, van Doorn HR, Ly VT, Le T, Cuomo CA. 2023. Impact of pathogen genetics on clinical phenotypes in a population of *Talaromyces marneffei* from Vietnam. *Genetics* 224:iyad100.
4. Muñoz JF, Gauthier GM, Desjardins CA, Gallo JE, Holder J, Sullivan TD, Marty AJ, Carmen JC, Chen Z, Ding L, Gujja S, Magrini V, Misas E, Mitreva M, Priest M, Saif S, Whiston EA, Young S, Zeng Q, Goldman WE, Mardis ER, Taylor JW, McEwen JG, Clay OK, Klein BS, Cuomo CA. 2015. The Dynamic Genome and Transcriptome of the Human Fungal Pathogen *Blastomyces* and Close Relative *Emmonsia*. *PLoS Genet* 11:e1005493.
5. Muñoz JF, Gallo JE, Misas E, Priest M, Imamovic A, Young S, Zeng Q, Clay OK, McEwen JG, Cuomo CA. 2014. Genome update of the dimorphic human pathogenic fungi causing paracoccidioidomycosis. *PLoS Negl Trop Dis* 8:e3348.
6. Hudson J, Budvytiene I, Banaei N. 2025. Diagnostic stewardship applied to targeted fungal sequencing. *J Clin Microbiol* 63:e00896-25.
7. Chuvochina M, Gerken J, Frentrup M, Sandikci Y, Goldmann R, Freese HM, Göker M, Sikorski J, Yarza P, Quast C, Peplies J, Glöckner FO, Reimer LC. 2026. SILVA in 2026: a global core biodata resource for rRNA within the DSMZ digital diversity. *Nucleic Acids Research* 54:D334–D341.

8. Bhattacharyya RP, Walker M, Boykin R, Son SS, Liu J, Hachey AC, Ma P, Wu L, Choi K, Cummins KC, Benson M, Skerry J, Ryu H, Wong SY, Goldberg MB, Han J, Pierce VM, Cosimi LA, Shoresh N, Livny J, Beechem J, Hung DT. 2019. Rapid identification and phylogenetic classification of diverse bacterial pathogens in a multiplexed hybridization assay targeting ribosomal RNA. *Sci Rep* 9:4516.

9. Geiss GK, Bumgarner RE, Birditt B, Dahl T, Dowidar N, Dunaway DL, Fell HP, Ferree S, George RD, Grogan T, James JJ, Maysuria M, Mitton JD, Oliveri P, Osborn JL, Peng T, Ratcliffe AL, Webster PJ, Davidson EH, Hood L, Dimitrov K. 2008. Direct multiplexed measurement of gene expression with color-coded probe pairs. *Nat Biotechnol* 26:317–325.
